# Identification and characteristics of patients with potential difficult-to-treat psoriatic arthritis: exploratory analyses of the Greek PsA registry

**DOI:** 10.1093/rheumatology/keae263

**Published:** 2024-05-17

**Authors:** Konstantinos D Vassilakis, Charalampos Papagoras, Nikolaos Fytanidis, Sousana Gazi, Evangelia Mole, Michael Krikelis, Paraskevi V Voulgari, Evripidis Kaltsonoudis, Nikolaos Koletsos, Dimitrios Boumpas, Pelagia Katsimpri, Dimitrios Katsifis-Nezis, Theodoros Dimitroulas, Nikolaos Kougkas, Maria Boutel, Petros P Sfikakis, Maria G Tektonidou, Chrysoula Gialouri, Dimitrios Bogdanos, Theodora Simopoulou, Christos Koutsianas, Evgenia Mavrea, Gkikas Katsifis, Konstantinos Kottas, Maria Konsta, Matthoula Tziafalia, Evangelia Kataxaki, Eleni Kalavri, Kalliopi Klavdianou, Eleftheria P Grika, Charalampos Sfontouris, Dimitrios Daoussis, George Iliopoulos, Ilias Bournazos, Dimitrios Karokis, Konstantinos Georganas, Dimos Patrikos, Dimitrios Vassilopoulos, George E Fragoulis

**Affiliations:** Joint Academic Rheumatology Program, First Department of Propedeutic and Internal Medicine, National and Kapodistrian University of Athens, Athens, Greece; First Department of Internal Medicine, University Hospital of Alexandroupolis, Democritus University of Thrace, Alexandroupolis, Greece; First Department of Internal Medicine, University Hospital of Alexandroupolis, Democritus University of Thrace, Alexandroupolis, Greece; Department of Rheumatology, KAT Hospital, Athens, Greece; Department of Rheumatology, KAT Hospital, Athens, Greece; Department of Rheumatology, KAT Hospital, Athens, Greece; Department of Rheumatology, School of Health Sciences, Faculty of Medicine, University of Ioannina, Ioannina, Greece; Department of Rheumatology, School of Health Sciences, Faculty of Medicine, University of Ioannina, Ioannina, Greece; Department of Rheumatology, School of Health Sciences, Faculty of Medicine, University of Ioannina, Ioannina, Greece; Joint Academic Rheumatology Program, 4th Department of Internal Medicine, Attikon University Hospital, National and Kapodistrian University of Athens Medical School, Athens, Greece; Joint Academic Rheumatology Program, 4th Department of Internal Medicine, Attikon University Hospital, National and Kapodistrian University of Athens Medical School, Athens, Greece; Joint Academic Rheumatology Program, 4th Department of Internal Medicine, Attikon University Hospital, National and Kapodistrian University of Athens Medical School, Athens, Greece; 4th Department of Medicine, Aristotle University, Thessaloniki, Greece; 4th Department of Medicine, Aristotle University, Thessaloniki, Greece; 4th Department of Medicine, Aristotle University, Thessaloniki, Greece; Joint Academic Rheumatology Program, First Department of Propedeutic and Internal Medicine, National and Kapodistrian University of Athens, Athens, Greece; Joint Academic Rheumatology Program, First Department of Propedeutic and Internal Medicine, National and Kapodistrian University of Athens, Athens, Greece; Joint Academic Rheumatology Program, First Department of Propedeutic and Internal Medicine, National and Kapodistrian University of Athens, Athens, Greece; Department of Rheumatology and Clinical Immunology, University of Thessaly, Larissa, Greece; Department of Rheumatology and Clinical Immunology, University of Thessaly, Larissa, Greece; Joint Academic Rheumatology Program, Clinical Immunology, Rheumatology unit, 2nd Department of Medicine and Laboratory, National and Kapodistrian University of Athens Medical School, General Hospital of Athens “Hippokration”, Athens, Greece; Joint Academic Rheumatology Program, Clinical Immunology, Rheumatology unit, 2nd Department of Medicine and Laboratory, National and Kapodistrian University of Athens Medical School, General Hospital of Athens “Hippokration”, Athens, Greece; Rheumatology Clinic, Naval Hospital of Athens, Athens, Greece; Rheumatology Clinic, Naval Hospital of Athens, Athens, Greece; Rheumatology Unit, Sismanoglio Hospital, Athens, Greece; Rheumatology Unit, Sismanoglio Hospital, Athens, Greece; Rheumatology Department, General Hospital Elefsinas Thriaseio, Athens, Greece; Department of Rheumatology, “Asklepieion” General Hospital, Athens, Greece; Department of Rheumatology, “Asklepieion” General Hospital, Athens, Greece; Department of Rheumatology, Evaggelismos Athens General Hospital, Athens, Greece; Department of Rheumatology, Evaggelismos Athens General Hospital, Athens, Greece; Department of Rheumatology, Patras University Hospital, University of Patras Medical School, Patras, Greece; Department of Rheumatology, Patras University Hospital, University of Patras Medical School, Patras, Greece; Private Practice, Athens, Greece; Private Practice, Athens, Greece; Private Practice, Athens, Greece; Private Practice, Athens, Greece; Joint Academic Rheumatology Program, Clinical Immunology, Rheumatology unit, 2nd Department of Medicine and Laboratory, National and Kapodistrian University of Athens Medical School, General Hospital of Athens “Hippokration”, Athens, Greece; Joint Academic Rheumatology Program, First Department of Propedeutic and Internal Medicine, National and Kapodistrian University of Athens, Athens, Greece; School of Infection and Immunity, University of Glasgow, Glasgow, UK

**Keywords:** psoriatic arthritis, difficult-to-treat (D2T), psoriasis, refractory, axial disease

## Abstract

**Objective:**

To present the characteristics of patients with potential difficult-to-treat (D2T) PsA.

**Methods:**

We used data from the Greek multicentre registry of PsA patients. D2T PsA was defined as follows: patients with at least 6 months’ disease duration, who have failed to at least one conventional synthetic DMARD and at least two biologic DMARDs/targeted synthetic DMARDs with a different mechanism of action and have either at least moderate disease activity (MODA) defined as DAPSA (Disease Activity index in PSoriatic Arthritis) >14, and/or are not at minimal disease activity (MDA). Demographic and clinical characteristics were compared between D2T and non-D2T PsA patients. In two sensitivity analyses, patients classified as D2T solely according to the MODA or MDA criterion were examined separately.

**Results:**

Among 467 patients included, 77 (16.5%) were considered D2T and 390 non-D2T PsA. Compared with non-D2T, patients with D2T PsA presented more commonly with extensive psoriasis (*P* < 0.0001) and were more likely to have higher BMI (*P* = 0.023) and a history of IBD (*P* = 0.026). In the MODA and MDA sensitivity analyses, 7.5% and 12.5% of patients were considered D2T, respectively. In both sensitivity analyses, extensive psoriasis was again identified as an independent variable for D2T PsA (*P* = 0.001 and *P* = 0.008, respectively). Moreover, female gender (*P* = 0.034) in the MODA analysis and axial disease (*P* = 0.040) in the MDA analysis were independent variables for D2T PsA.

**Conclusion:**

Despite the availability of therapies, D2T PsA is common in real-life cohorts of patients with PsA and extensive psoriasis. High BMI, female gender, axial disease and history of IBD were also associated with D2T PsA.

Rheumatology key messages

- In a real-life nationwide cohort, 10–15% of the PsA patients were ‘difficult-to-treat’ (D2T).
- Extensive psoriasis was consistently found to associate with D2T PsA.
- BMI, female gender, axial disease and IBD were also associated with D2T PsA.

## Introduction

PsA is an immune-mediated inflammatory disease, which falls under the umbrella of the spondyloarthritides (SpA). PsA displays a broad spectrum of musculoskeletal manifestations, including peripheral arthritis, enthesitis and dactylitis, while axial involvement is observed in about 20–50% of patients [1]. Psoriasis is usually present at diagnosis, although it may occasionally appear after the onset of the musculoskeletal symptoms [2]. Extra-musculoskeletal manifestations, such as uveitis and IBD, are also seen in PsA, although less commonly compared with other SpA. In addition, PsA may be accompanied by comorbidities, such as cardiovascular disease and mental health disorders, which add another level of complexity to the treatment of those patients [3, 4].

As our understanding of the pathophysiologic mechanisms of PsA improves, new therapeutic options arise, allowing for more personalized treatment approaches. Approved medications for PsA currently include conventional synthetic DMARDs (csDMARDs), biologic DMARDs (bDMARDs) targeting TNF-α, IL-12/23, IL-23 or IL-17, as well as targeted synthetic DMARDs (tsDMARDs), including JAK inhibitors and apremilast. Although treatment efficacy is difficult to measure due to the diversity of disease manifestations, it is usually expressed as improvement in one or more composite disease activity indices. Currently, the three most commonly used indices are DAPSA (Disease Activity index in PSoriatic Arthritis), PASDAS (PsA Disease Activity Score) and MDA (minimal disease activity).

Despite the availability of numerous therapeutic options, many patients with PsA display residual disease activity and fail to achieve remission or at least low disease activity. In a recent systematic review and meta-analysis, the prevalence of PsA patients achieving MDA in real-world studies and randomized clinical trials was assessed [5]. In cross-sectional studies, the overall prevalence of MDA was 35% (95% CI 30–41%), varying from 17% (95% CI 7–34%) in patients treated with csDMARDs to 57% (95% CI 41–71%) in those treated with TNF inhibitors [5].

In analogy to RA, therefore, the concept of difficult to treat (D2T) PsA has arisen in the literature [6]. Relevant definitions are lacking but are underway by EULAR and GRAPPA (Group for Research and Assessment of Psoriasis and Psoriatic Arthritis) [7]. In this context, an international survey was carried out [8] assessing rheumatologists’ view on terminology and definition of D2T PsA. The questionnaire was answered by 243 rheumatologists worldwide, most (35%) of which considered that D2T status should be defined as failure of at least two bDMARDs with different mechanism of action, while regarding treatment targets ‘low disease activity’ was preferred compared with ‘remission’. On the other hand, there are concerns over whether these patients are truly D2T or whether the term ‘refractory’ might be used as an alternative [9], encompassing the comorbidities like depression/FM that often come with PsA.

Herein, utilizing data from a large, multicentre registry of patients with PsA, we examined the characteristics of PsA patients who were potential D2T. For simplicity, we used a definition that was based on the paradigm set by the respective EULAR project for RA [6].

## Methods

Data were retrieved from the multicentre registry of patients with PsA (CASPAR criteria) which is ongoing in Greece, under the auspices of the Greek Rheumatology Society. For this study, we analysed cross-sectionally patients who were assessed in the participating rheumatology clinics/practices in Greece between 1 January 2022 and 31 December 2022. The design of the registry is described elsewhere [2]. In brief, the following characteristics were recorded on an electronic platform: demographics (including working and educational status); time of follow-up (time from disease diagnosis to the time of assessment); disease clinical and laboratory characteristics, including joint counts, axial involvement (defined as inflammatory back pain accompanied by imaging findings in X-rays or MRI), enthesitis, dactylitis, skin [expressed as body surface area (BSA)] and nail disease at the time of diagnosis as well as over the disease course, current DAPSA, comorbidities (coronary heart disease, stroke, hypercholesterolemia, diabetes mellitus, hypertension, hyperuricemia, depression, osteoporosis) defined as described [2]; as well as past and current anti-rheumatic treatments.

For this project, we considered as potential D2T PsA patients who had at least 6 months of disease duration, have failed to respond to at least one csDMARDs (unless contraindicated) and at least two bDMARDs/tsDMARDs (except from apremilast) with a different mechanism of action, and had either at least moderate disease activity (MODA—defined as DAPSA >14), and/or were not in MDA (‘main definition’) at the time of their assessment (Table 1). For this analysis, we excluded patients whose disease activity by DAPSA was <14 and MDA was not available, or patients who were in MDA, but DAPSA was not available.

**Table 1.** Definitions for potential D2T PsA patients, used in this study. In all definitions, patients need to have at least 6 months of disease duration

Main definition	(i) Failure to respond to at least one csDMARDs^b^ and at least two bDMARDs/tsDMARDs^a^ with a different mechanism of action
	AND
	(ii) Having at least moderate disease activity (MODA—defined as DAPSA >14), and/or not being MDA
Alternate definition #1 (MODA definition 1)	(ii) Failure to respond to at least one csDMARDs^b^ and at least two bDMARDs/tsDMARDs^a^ with a different mechanism of action
	AND
	(ii) DAPSA >14
Alternate definition #2 (MDA definition 2)	(i) Failure to respond to at least one csDMARDs^b^ and at least two different b/tsDMARDs^a^ with a different mechanism of action
	AND
	(ii) Not being in MDA

#1: irrespective of MDA status; #2; irrespective of DAPSA value. ^a^Except from apremilast. ^b^Unless contraindicated. bDMARD: biologic DMARD; csDMARD: conventional synthetic DMARD; D2T: difficult to treat; DAPSA: Disease Activity index in PSoriatic Arthritis; MDA: minimal disease activity; MODA: moderate disease activity; tsDMARDs: targeted synthetic DMARD.

Two sensitivity analyses were conducted based on alternative definitions of D2T PsA. According to the first alternative definition, patients had DAPSA >14 (‘MODA definition’—irrespective of MDA status), and they had failed at least one csDMARDs (unless contraindicated) and at least two bDMARDs/tsDMARDs (Table 1). According to the second alternative definition, D2T patients should not have been in MDA (‘MDA definition’—irrespective of DAPSA value), and again had been unsuccessfully treated with at least one csDMARDs (unless contraindicated) and at least two different b/tsDMARDs (Table 1).

For statistical analysis, categorical characteristics were compared with two-sided χ^2^, while numerical values were compared with unpaired *t*-test or Mann–Whitney test for parametric or non-parametric variables, respectively. Normal distribution was checked with Kolmogorov–Smirnov test. Multivariable binomial regression analysis was conducted, setting age and sex and all variables significantly different in the univariable analyses as independent variables, while D2T status was the dependent variable. Results were expressed as odds ratios (OR) along with 95% CI. Statistical significance was considered for *P-*values <0.05. GraphPad Prism 5.00 (GraphPad Software, Inc., Boston, MA, USA) and SPSS 24.0 (SPSS software, Chicago, IL, USA) were used.

Institutional Review Board approval was provided by the Joint Rheumatology Program (‘Laiko Hospital’ No.: 780/2021) and by the local institutional boards of participating centres. All patients provided written informed consent.

## Results

### Cohort description, demographics

**Figure 1.**
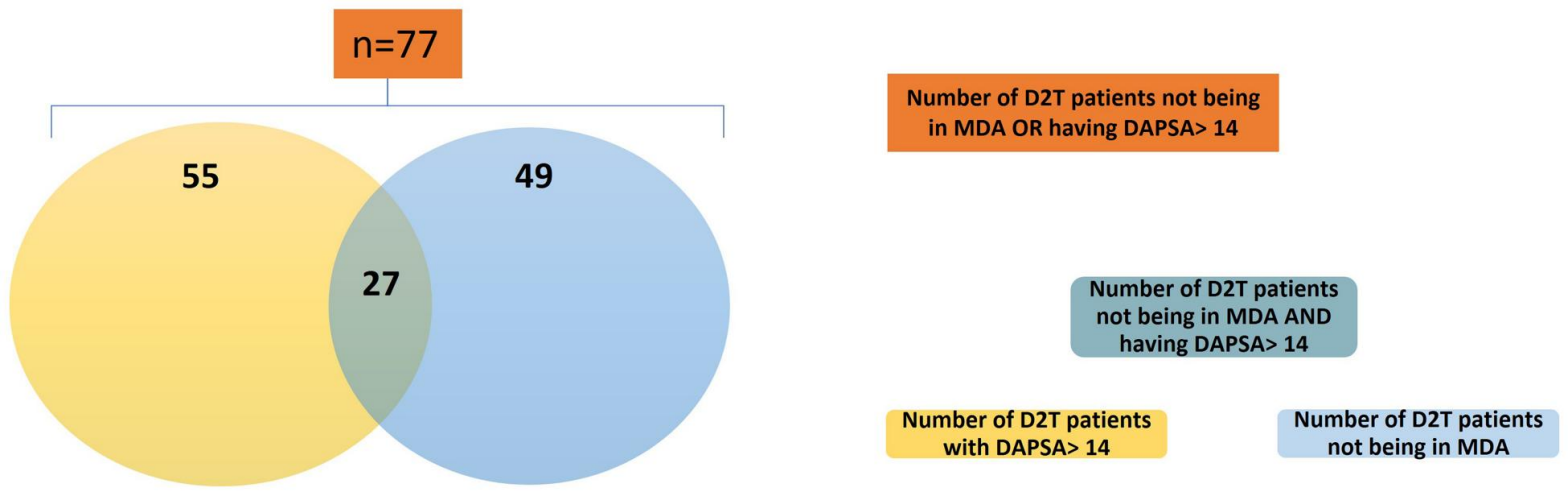
Schematic representation of the D2T patients included in the study. MDA: minimal disease activity; D2T: difficult-to-treat

Out of a total of 738 registered patients, in the analysis using the ‘main definition’, 467 patients were included. Among them, 77 (16.5%) were considered D2T (Fig. 1) and 390 (83.5%) non-D2T patients (Table 2). The mean age (s.d.) was 54.1 (12.4) years for the D2T and 56.8 (11.8) years for the non-D2T group, respectively. As shown in Table 2, patients in the D2T group were more commonly female, current smokers and had higher mean BMI. Additionally, patients with D2T PsA were more frequently unemployed. In contrast, age, family history, disease duration and educational status did not differ between subgroups (Table 2). From the D2T patients, 27, 18, 22 and 10 had received two, three, four or five (or more) bDMARDs/tsDMARDs, respectively. The type of ever-received b/tsDMARDs is depicted in Supplementary Table S1, available at *Rheumatology* online.

**Table 2.** Demographics of D2T and non-D2T PsA patients according to the main definition

Features	D2T (*N* = 77)	Non-D2T (*N* = 390)	*P*-values
Age, years, mean (s.d.)	54.1 (12.36)	56.8 (11.85)	0.129
Females, *n* (%)	55 (71)	223 (57)	**0.022**
BMI, kg/m^2^, mean (s.d.)	31.77 (7.06)	28.95 (5.48)	**0.002**
Smoking, *n* (%)	31/74 (42)	110/375 (29)	**0.040**
Disease duration, months, mean (s.d.)	113.5 (86.60)	119.5 (93.91)	0.792
Family history of axSpA, *n* (%)	3 (4)	11 (3)	0.712
Family history of PsA, *n* (%)	4 (5)	29 (7)	0.629
Family history of psoriasis, *n* (%)	22 (29)	112 (29)	1.000
Family history of IBD, *n* (%)	0/77 (0)	5/390 (1)	1.000
Employment status, *n* (%)			**0.009**
Unemployed	14/71 (20)	33/385 (9)	
Employed	36/71 (51)	244/385 (63)	**0.047**
Retired	21/71 (29)	108/385 (28)	0.776
Educational status, *n* (%)	9/64 (14)	47/379 (12)	0.686
Primary			
Secondary	37/64 (58)	227/379 (60)	0.784
Tertiary	18/64 (28)	105/379 (28)	1.000

D2T: difficult-to-treat; axSpA: axial SpA. Significant (<0.05) *P*-values are shown in bold.

### Clinical manifestations and comorbidities

At the time of PsA diagnosis, univariable analyses showed that D2T patients had less frequently dactylitis compared with non-D2T patients, while in the D2T group, psoriasis, as assessed by BSA index, was more severe (Table 3). As regards the rest of the clinical characteristics and comorbidities, patients in the D2T group exhibited more frequently axial involvement, nail disease ever and IBD ever, while depression was also more common compared with those with non-D2T PsA (Tables 3 and 4). Further multivariable binomial regression showed that D2T patients, compared with the non-D2T group, were more likely to have extensive psoriasis, defined as BSA >3% (OR 5.05, 95% CI 2.22–11.47, *P* < 0.0001) at diagnosis. In addition, it was shown that D2T patients displayed more frequently a greater BMI (OR 1.07, 95% CI 1.01–1.13, *P* = 0.023) and IBD ever (OR 1.22, 95% CI 1.25–31.06, *P* = 0.026).

**Table 3.** Clinical manifestations at diagnosis and at any time following the diagnosis of PsA in D2T and non-D2T patients, according to the main definition

Features	D2T (*N* = 77)	Non-D2T (*N* = 390)	*P*-values
Characteristics at diagnosis, *n* (%)			
Peripheral arthritis	61 (79)	330 (85)	0.240
Axial disease	18 ((23)	56 (14)	0.059
Enthesitis	17/75 (23)	123/381 (32)	0.103
Dactylitis	10/75 (13)	92/380 (24)	**0.048**
Nail	30/72 (42)	127/378 (34)	0.224
Uveitis	0/72 (0)	8/375 (2)	0.365
BSA <3	34/53 (64)	320/358 (89)	**0.0001**
BSA 3–10	15/53 (28)	34/358 (9)	**0.0004**
BSA >10	4/53 (8)	4/358 (1)	0.0117
Characteristics ever, *n* (%)			
Axial disease	29/77 (38)	81/390 (21)	**0.003**
Enthesitis	43/75 (57)	184/381 (48)	0.166
Dactylitis	26/75 (35)	140/380 (37)	0.793
Nail	41/72 (57)	163/378 (43)	**0.038**
Uveitis	2/72 (3)	13/375 (3)	1.000
IBD	5/73 (7)	5/380 (1)	**0.012**

D2T: difficult-to-treat; BSA: body surface area; DAPSA: Disease Activity index in PSoriatic Arthritis. Significant (<0.05) *P*-values are shown in bold.

**Table 4.** Comorbidities of D2T and non-D2T PsA patients, according to the main definition

Features	D2T (*n* = 77), *n* (%)	Non-D2T (*n* = 390), *n* (%)	*P-*values
CHD	2/69 (3)	23/372 (6)	0.399
Stroke	1/67 (1)	5/372 (1)	1.000
MACE	2/67(3)	27/372 (7)	0.285
Hypercholesterolemia	30/68 (44)	159/363 (44)	1.000
Diabetes mellitus	15/71 (21)	60/373 (16)	0.302
Hypertension	32/73 (44)	189/374 (51)	0.309
Hyperuricemia	7/69 (10)	59/367 (16)	0.272
Depression	27/67 (40)	90/370 (24)	**0.010**
Osteoporosis	5/69 (7)	47/366 (13)	0.228
Past/current neoplasia	2/69 (3)	17/317 (5)	0.546
Latent tuberculosis	10/51 (20)	37/304 (12)	0.178

D2T: difficult-to-treat; CHD: coronary heart disease; MACE: major adverse cardiovascular events. Significant (<0.05) *P*-values are shown in bold.

### D2T definition based on MODA (DAPSA)

Among the total of 738 patients in the registry, 728 patients were included in this sensitivity analysis (MODA definition). Fifty-five (7.55%) were considered D2T (Fig. 1) and 673 (92.45%) non-D2T. Mean age was 54.3 years (s.d. 12.2) for the D2T group and 56.6 years (s.d. 12.2) for the non-D2T group (*P* = 0.211). Patients in the D2T group were more commonly female (78% *vs* 52%, *P* = 0.0001) and current smokers (49% *vs* 27%, *P* = 0.001), exhibited higher BMI [mean (s.d.) 31.8 (7.6) *vs* 28.8 (5.7) kg/m^2^, *P* = 0.004] and were more frequently unemployed (24% *vs* 7%, *P* = 0.001) (Supplementary Table S2, available at *Rheumatology* online).

Univariable analysis also showed that, at diagnosis, D2T patients had more extensive psoriasis compared with non-D2T ones (BSA <3 in 61% of D2T *vs* 86% in non-D2T, *P* = 0.001). Moreover, in the D2T group, axial disease at diagnosis (25% *vs* 12% *P* = 0.009) and ever since PsA diagnosis was more common (40% *vs* 19%, *P* = 0.001), as well as nail involvement ever (62% *vs* 45% *P* = 0.024) (Supplementary Table S3, available at *Rheumatology* online). The prevalence of depression (49% *vs* 22%, *P* = 0.001) was also higher in the D2T group (Supplementary Table S4, available at *Rheumatology* online). In the multivariable analysis it was shown that BSA >3% at diagnosis (OR 4.89, 95% CI 1.89 = 12.64, *P* = 0.001) and female gender (OR 3.03, 95% CI 1.08–8.47, *P* = 0.034) were independently associated with D2T PsA.

### D2T definition based on MDA

When we used the MDA definition, 393 out of 738 patients were included in the analysis. Forty-nine (12.47%) were considered D2T (Fig. 1) and 344 (87.53%) non-D2T. Mean age (s.d.) was 53.1 (12.8) years for the D2T group and 56.8 (12.1) years for the non-D2T group (*P* = 0.056). D2T patients were more commonly female (69% *vs* 55%, *P* = 0.064) and were more likely to be unemployed (19% *vs* 8% *P* = 0.0029) (Supplementary Table S5, available at *Rheumatology* online). At diagnosis, extensive psoriasis (BSA >3%) was common in the D2T group (*P* = 0.001), whereas it was also more likely for these patients to have axial (37% *vs* 20%, *P* = 0.016) and nail (61% *vs* 44%, *P* = 0.032) involvement at some point during the disease course (Supplementary Table S6, available at *Rheumatology* online). Regarding comorbidities, hypertension (75% *vs* 47%, *P* = 0.002) and history of past or current neoplasia (15% *vs* 5%, *P* = 0.020) were more common in the D2T group (Supplementary Table S7, available at *Rheumatology* online). Multivariable binomial regression showed that BSA >3% at diagnosis (OR 3.28, 95% CI 1.36–7.90, *P* = 0.008) and axial disease ever (OR 2.23, 95% CI 1.04–4.77, *P* = 0.040) were independently associated with D2T PsA.

## Discussion

In this nationwide multicentre analysis, we found that, based on our initial D2T PsA definition (main definition), which is a modification of the previously proposed D2T RA description [6], one in six patients with PsA had potential D2T disease (16.5%). Conducting sensitivity analyses however, with two alternate definitions using MODA or MDA as an outcome, the prevalence of D2T PsA was 7.5% and 12.5%, respectively.

In our study, the extent of psoriasis at diagnosis was associated with a D2T status in all analyses (i.e. main, MODA and MDA), indicating the importance of psoriasis severity in achieving joint disease remission by different therapies. These findings are in accordance with an Italian retrospective study, which exhibited that D2T patients had a significantly higher BSA score at baseline compared with the control group [10]. Similarly, a French retrospective study enrolling 150 patients showed that the discontinuation of bDMARDs due to a poor dermatological response was associated with D2T PsA [11].

Regarding obesity/high BMI, our results are in agreement with one of the aforementioned studies [10] which also shows that BMI findings are usually higher in D2T PsA. Besides, it is well known that in patients with inflammatory arthritis, response to b/tsDMARDs is suboptimal [12, 13].

IBD was also identified as one of the parameters associated with D2T PsA, although CIs were quite large owing to the low number of cases. This is not unexpected since the frequency of IBD is <5% in the setting of PsA [2]. Also, occurrence of IBD narrows the therapeutic options for PsA, as IL-17 inhibitors and etanercept are not used in active bowel involvement [14].

When it comes to comorbidities, we found that in univariable (but not in multivariable analyses) the occurrence of depression was higher in the D2T group. This was not an unexpected finding, as depression is a quite common comorbidity in the setting of PsA [15, 16] and can affect the indices we use for assessing PsA disease activity [13, 17]. This is also in line with the Italian study [10] which found FM to be more common in D2T patients. Besides, on clinical grounds it is sometimes difficult to distinguish depression from FM, which has also been found to have a negative impact on patient-reported outcomes [15, 18].

Additionally, in the sensitivity analysis using only MODA as a criterion (MODA definition), it was shown that D2T patients were more commonly female. These findings agree with published data [9, 19], also systematically reviewed [20], showing that female patients display a higher disease burden and worse treatment outcomes compared with males with PsA.

In the sensitivity analysis using only MDA as a criterion (MDA definition), it was shown that D2T patients were more likely to have axial involvement throughout disease course. Axial disease at baseline was also found more frequently in D2T patients in the French study [11]. It should be mentioned that the definition of axial involvement in PsA differs among studies [1, 21–23] and a broadly accepted definition is still awaited [24].

The definition of D2T PsA is quite challenging, as PsA itself is a multifactorial and complex entity [9]. In the scoping literature review, performed by GRAPPA for D2T PsA [7], it was noted that there was conflict of opinions regarding to the definition of inadequate treatment response and active disease. In daily clinical practice, disease activity indices such as MDA and DAPSA are used to assess treatment response and therefore they may be helpful in identifying D2T individual patients. However, these indices cannot reflect the global burden of PsA, as all of them present strengths, weaknesses and disparities [13]. Thus, the lack of a widely recognized PsA activity index, combined with the highly heterogeneous nature of the disease, makes it even more difficult to set a definition, whereas at the same time it raises further concerns about the management of these patients. Of note, proposed adverse prognostic factors [25] for PsA outcomes were not associated with D2T PsA in our or other studies, apart from radiographic damage which was identified in the French study. This indicates the complexity of PsA in terms of phenotyping [26], but also in measuring disease activity [13]. Along these lines, the definition of D2T PsA might not be identical to that used for RA [27] as these two entities have significant differences. From this point of view, we have not included treatment with glucocorticoids as a criterion for D2T PsA, as glucocorticoids are not routinely used in the treatment of PsA. It might, however, be worth noting that current treatment with prednisolone ≥7.5 mg/day was more common in D2T patients compared with the non-D2T (33% *vs* 15% for the main definition; 30% *vs* 8% for the MODA definition; 20% *vs* 10% for the MDA definition). Lastly, it is important to note that the definition of D2T applied in this study has not achieved consensus within the international community. Thus, it is often difficult to fully understand the difference between refractory PsA, which is characterized by resistant inflammation at a clinical, imaging or laboratory level. On the other hand, it has been suggested that D2T PsA encompasses the comorbidities like depression/FM that often come with this disease as well as other parameters like pain and quality of life [9]. Herein, for simplicity but also to be in the same page with other groups [10, 11] we opted to use the term ‘D2T’. We acknowledge however that a definition as such will be agreed by international organizations like EULAR and GRAPPA.

We acknowledge that our study has certain limitations. First, as mentioned, definitions for D2T PsA are lacking. This might explain the discrepancies between our results and other studies [10, 11]. Further to the EULAR D2T definitions for RA, similar initiatives are expected for PsA from EULAR and GRAPPA. Second, comorbidities, like depression, are reported based on treatment received rather than a specific questionnaire. It cannot thus be excluded that FM, which has been associated with DT2 PsA [10] could be the culprit for some of the symptomatology. It must be mentioned, however, that the same methodology has been used in other studies from our group [16] and the percentages of depression reported were quite similar to those derived from studies using depression-specific questionnaires [3] and/or respective meta-analyses [28]. Third, we do not have data about whether bDMARDs/tsDMARDs were co-administered with csDMARDs and how this could have impacted their efficacy. Of note, frequency of taken-ever csDMARDs was not different between D2T and non-D2T (across various definitions) apart from LEF and SSZ, which were more often used in D2T patients (main definition; Supplementary Tables S8–S10, available at *Rheumatology* online). Fourth, in our definition of D2T PsA, indices like HAQ quality of life were not included as a considerable amount of data were missing for this parameter. Finally, considering that D2T PsA is a dynamic state, time, in terms of monitoring, plays a crucial role in what we eventually call D2T. A cut-off point of an at least 6 months’ disease duration was considered for this study. However, this strategy guarantees the D2T status only within the monitoring duration. Thus, we could not know whether some individuals currently considered as D2T, would potentially become non-D2T in the future or vice versa.

On the other hand, a strength of our study is the large number of patients. In fact, this is the largest study in the field with the data derived from a registry that was designed to capture the characteristics of PsA in Greece. Given that the present study is a secondary analysis of the Greek PsA registry, there are some missing data, but these are typically <10%. Of note, it is a multicentre study with a wide representation of different hospitals across the country. Therefore, selection bias is likely avoided.

In conclusion, our exploratory analysis using a large database of patients with PsA and three different definitions of D2T PsA, demonstrated that up to one every six patients has D2T PsA in real-life settings, whereas certain patient (female gender, obesity) and disease (extensive skin disease, axial involvement, history of IBD) characteristics were associated with its occurrence. These findings are of help in the ongoing effort for better defining D2T PsA and more importantly for designing management strategies to decrease its incidence.

## Supplementary Material

keae263_Supplementary_Data

## Data Availability

All data are available upon reasonable request to the corresponding author.
