# Supplementary material for: Identification and characteristics of patients with potential difficult-to-treat psoriatic arthritis: exploratory analyses of the Greek PsA registry
Source: Rheumatology (Oxford). 2024 May 17;63(9):2427–32. doi: 10.1093/rheumatology/keae263 (PMC11371370; doi:10.1093/rheumatology/keae263)
Supplement: keae263_Supplementary_Data [file keae263_supplementary_data.docx]

**Supplementary Tables**

| **Features** | **D2T**  **(n=77)** | **Non-D2T**  **(n=390)** |
| --- | --- | --- |
| *TNF inhibitors* | | |
| Adalimumab | 57 (74%) | 114 (29%) |
| Certolizumab | 20 (26%) | 40 (10%) |
| Golimumab | 13 (17%) | 23 (6%) |
| Infliximab | 27 (35%) | 81 (21%) |
| Etanercept | 24 (31%) | 74 (19%) |
| *IL-12/IL-23 inhibitors* | | |
| Ustekinumab | 25 (32%) | 24 (6%) |
| *IL-17A inhibitors* | | |
| Ixekizumab | 16 (21%) | 5 (1%) |
| Secukinumab | 52 (68%) | 44 (11%) |
| *IL-17RA inhibitors* | | |
| Brodalumab | 0 (0%) | 1 (<1%) |
| *Co-stimulation inhibitor* | | |
| Abatacept | 2 (3%) | 2 (<1%) |
| *JAK inhibitors* | | |
| Tofacitinib | 11 (14%) | 4 (1%) |
| Baricitinib | 1 (1%) | 0 (0%) |

**Supplementary Table-1:** Targeted synthetic and biologic DMARDs ever received in D2T and non-D2T PsA individuals

D2T (Difficult-to-treat), TNF (Tumor necrosis factor), IL (Interleukin), JAK (Janus Kinase), DMARDs (Disease

modifying antirheumatic drugs), PsA (Psoriatic arthritis)

| **Features** | **D2T**  **(n=55)** | **Non-D2T**  **(n=673)** | **P values** |
| --- | --- | --- | --- |
| **Demographics** | | | |
| Age, years, mean (SD) | 54.35 (12.18) | 56.63 (12.23) | 0.211 |
| Females, n (%) | 43 (78%) | 352 (52%) | **0.001** |
| BMI, kg/m^2^, mean (SD) | 31.78 (7.58) | 28.76 (5.73) | **0.004** |
| Smoking, n (%) | 26/53 (49%) | 173/639 (27%) | **0.001** |
| Disease duration, months, mean (SD) | 116.1 (91.78) | 124.2 (95.39) | 0.550 |
| Family history of axSpA, n (%) | 2 (4%) | 17 (3%) | 0.648 |
| Family history of PsA, n (%) | 3 (5%) | 40 (6%) | 1.000 |
| Family history of psoriasis, n (%) | 15 (27%) | 199 (30%) | 0.877 |
| Family history of IBD, n (%) | 0 (0%) | 8 (1%) | 1.000 |
| Employment status, n (%) | Unemployed: 12/50 (24%)  Employed: 21/50 (42%)  Retired: 17/50 (34%) | Unemployed: 48/655 (7%)  Employed: 417/655 (64%)  Retired: 190/655 (29%) | **0.001**  **0.004**  0.519 |
| Educational status, n (%) | Primary: 7/43 (16%)  Secondary: 24/43 (56%)  Tertiary: 12/43 (28%) | Primary: 80/642 (12%)  Secondary: 368/642 (57%)  Tertiary: 194/642 (30%) | 0.476  0.874  0.864 |

**Supplementary Table-2:** Demographics of D2T and non-D2T PsA according to the MODA definition

D2T (Difficult-to-treat), SD (Standard Deviation), BMI (Body mass index), axSpA (axial spondyloarthritis), PsA (psoriatic arthritis), IBD (inflammatory bowel disease), MODA moderate disease activity

| **Features** | **D2T**  **(n=55 )** | | **Non-D2T**  **(n=673)** | | **P values** | |
| --- | --- | --- | --- | --- | --- | --- |
| **Characteristics at diagnosis** | | | | | | |
| Peripheral arthritis, n (%) | | 44 (80%) | | 545 (81%) | | 0.859 |
| Axial disease, n (%) | | 14 (25%) | | 79 (12%) | | **0.009** |
| Enthesitis, n (%) | | 12 (22%) | | 179 (27%) | | 0.535 |
| Dactylitis, n (%) | | 8 (15%) | | 129 (19%) | | 0.476 |
| Nail, n (%) | | 23 (42%) | | 211 (31%) | | 0.132 |
| Uveitis, n (%) | | 0 (0%) | | 11 (2%) | | 1.000 |
| BSA <3, n (%) | | 19/31 (61%) | | 338/386 (86%) | | **0.001** |
| BSA 3-10, n (%) | | 9/31 (29%) | | 43/386 (11%) | | **0.008** |
| BSA >10, n (%) | | 3/31 (10%) | | 5/386 (1%) | | **0.016** |
| **Characteristics ever** | | | | | | |
| Axial disease, n (%) | | 22 (40%) | | 131 (19%) | | **0.001** |
| Enthesitis, n (%) | | 35 (64%) | | 347 (52%) | | 0.093 |
| Dactylitis, n (%) | | 22 (40%) | | 230 (34%) | | 0.380 |
| Nail, n (%) | | 34 (62%) | | 305 (45%) | | **0.024** |
| Uveitis, n (%) | | 5 (9%) | | 56 (8%) | | 0.800 |
| IBD, n (%) | | 6 (11%) | | 58 (9%) | | 0.617 |

**Supplementary Table-3:** Clinical manifestations at diagnosis and at any time of D2T and non-D2T PsA patients according to the MODA definition

D2T (Difficult-to-treat), SD (Standard Deviation), BSA (body surface area), DAPSA (disease activity index for psoriatic arthritis), IBD (inflammatory bowel disease)

| **Features** | **D2T**  **(n=55)** | **Non-D2T**  **(n=673)** | **P values** |
| --- | --- | --- | --- |
| CHD, n (%) | 2/48 (4%) | 37/637 (6%) | 1.000 |
| Stroke, n (%) | 0/47 (0%) | 9/634 (1%) | 1.000 |
| MACE, n (%) | 1/47 (2%) | 43/634 (7%) | 0.352 |
| Hypercholesterolemia, n (%) | 18/46 (39%) | 279/628 (44%) | 0.540 |
| Diabetes Mellitus, n (%) | 10/49 (20%) | 103/641 (16%) | 0.424 |
| Hypertension, n (%) | 22/51 (43%) | 313/646 (48%) | 0.561 |
| Hyperuricemia, n (%) | 4/48 (8%) | 112/635 (18%) | 0.112 |
| Depression, n (%) | 23/47 (49%) | 142/635 (22%) | **0.001** |
| Osteoporosis, n (%) | 5/48 (10%) | 77/631 (12%) | 1.000 |
| Past/current neoplasia, n (%) | 2/48 (4%) | 25/528 (5%) | 1.000 |
| Latent tuberculosis, n (%) | 6/34 (18%) | 80/486 (16%) | 0.813 |

**Supplementary Table-4:** Comorbidities of D2T and non-D2T PsA patients according to the MODA definition D2T (Difficult-to-treat), CHD (coronary heart disease), MACE (major adverse cardiovascular events)

| **Features** | **D2T**  **(n=49)** | **Non-D2T**  **(n=344)** | **P values** |
| --- | --- | --- | --- |
| **Demographics** | | | |
| Age, years, mean (SD) | 53.18 (12.83) | 56.76 (12.11) | 0.056 |
| Female Gender, n (%) | 34 (69%) | 188 (55%) | 0.064 |
| BMI, kg/m^2^, mean (SD) | 28.76 (4.82) | 29.28 (5.70) | 0.877 |
| Smoking, n (%) | 8/47 (17%) | 99/333 (30%) | 0.083 |
| Disease duration, months, mean (SD) | 105.4 (75.70) | 121.7 (95.59) | 0.499 |
| Family history of axSpA, n (%) | 3 (6%) | 10 (3%) | 0.212 |
| Family history of PsA, n (%) | 3 (6%) | 26 (8%) | 1.000 |
| Family history of psoriasis, n (%) | 16 (33%) | 102 (30%) | 0.739 |
| Family history of IBD, n (%) | 0 (0%) | 5 (1%) | 1.000 |
| Employment status, n (%) | Unemployed: 9/47 (19%)  Employed: 25/47 (53%)  Retired: 13/47 (28%) | Unemployed: 28/340 (8%)  Employed: 217/340 (64%)  Retired: 95/340 (28%) | **0.029**  0.198  1.000 |
| Educational status, n (%) | Primary: 7/46 (15%)  Secondary: 27/46 (59%)  Tertiary: 12/46 (26%) | Primary: 43/338 (13%)  Secondary: 205/338 (61%)  Tertiary: 90/338 (27%) | 0.641  0.873  1.000 |

**Supplementary Table-5:** Demographics of D2T and non-D2T PsA according to the MDA definition

D2T (Difficult-to-treat), SD (Standard Deviation), BMI (Body mass index), axSpA (axial spondyloarthritis), PsA (psoriatic arthritis), IBD (inflammatory bowel disease)

| **Features** | **D2T**  **(n=49)** | | **Non-D2T**  **(n=344)** | | **P values** | |
| --- | --- | --- | --- | --- | --- | --- |
| **Characteristics at diagnosis** | | | | | | |
| Peripheral arthritis, n (%) | | 39 (80%) | | 290 (84%) | | 0.410 |
| Axial disease, n (%) | | 10 (20%) | | 51 (15%) | | 0.298 |
| Enthesitis, n (%) | | 12 (24%) | | 111 (32%) | | 0.325 |
| Dactylitis, n (%) | | 7 (14%) | | 87 (25%) | | 0.108 |
| Nail, n (%) | | 22 (45%) | | 115 (33%) | | 0.148 |
| Uveitis, n (%) | | 0 (0%) | | 8 (2%) | | 0.603 |
| BSA <3, n (%) | | 32 (65%) | | 308 (90%) | | **0.001** |
| BSA 3-10, n (%) | | 14 (29%) | | 32 (9%) | | **0.001** |
| BSA >10, n (%) | | 3 (6%) | | 4 (1%) | | **0.044** |
| **Characteristics ever** | | | | | | |
| Axial disease, n (%) | | 18 (37%) | | 70 (20%) | | **0.016** |
| Enthesitis, n (%) | | 28 (57%) | | 168 (49%) | | 0.289 |
| Dactylitis, n (%) | | 18 (37%) | | 134 (39%) | | 0.876 |
| Nail, n (%) | | 30 (61%) | | 153 (44%) | | **0.032** |
| Uveitis, n (%) | | 4 (8%) | | 18 (5%) | | 0.501 |
| IBD, n (%) | | 5 (10%) | | 19/(6%) | | 0.202 |

**Supplementary Table-6:** Clinical manifestations at diagnosis and present at any time in D2T and non-D2T PsA patients according to the MDA definition

D2T (Difficult-to-treat), SD (Standard Deviation), BSA (body surface area), DAPSA (disease activity index for psoriatic arthritis), IBD (inflammatory bowel disease)

| **Features** | **D2T**  **(n=49)** | | **Non-D2T**  **(n=344)** | | **P values** | |
| --- | --- | --- | --- | --- | --- | --- |
| **Comorbidities** | | | | | | |
| CHD, n (%) | | 4/47 (9%) | | 19/337 (6%) | | 0.506 |
| Stroke, n (%) | | 1/47 (2%) | | 5/336 (1%) | | 0.547 |
| MACE, n (%) | | 5/47 (11%) | | 23/336 (7%) | | 0.366 |
| Hypercholesterolemia, n (%) | | 24/47 (51%) | | 140/326 (43%) | | 0.346 |
| Diabetes Mellitus, n (%) | | 12/48 (25%) | | 46/336 (14%) | | 0.052 |
| Hypertension, n (%) | | 33/46 (72%) | | 160/339 (47%) | | **0.002** |
| Hyperuricemia, n (%) | | 12/47 (26%) | | 50/331 (15%) | | 0.090 |
| Depression , n (%) | | 11/47 (23%) | | 83/331 (25%) | | 1.000 |
| Osteoporosis, n (%) | | 8/47 (17%) | | 36/332 (11%) | | 0.224 |
| Past/current neoplasia, n (%) | | 6/41 (15%) | | 13/287 (5%) | | **0.020** |
| Latent tuberculosis, n (%) | | 5/36 (14%) | | 32/283 (11%) | | 0.587 |

**Supplementary Table-7:** Comorbidities of D2T and non-D2T PsA patients according to the MDA definition

D2T (Difficult-to-treat), CHD (coronary heart disease), MACE (major adverse cardiovascular events)

| **Features** | **D2T**  **(n=77)** | **Non-D2T**  **(n=390)** | **p-value** |
| --- | --- | --- | --- |
| Methotrexate | 64 (83%) | 334 (88%) | 0.598 |
| Leflunomide | 26 (34%) | 85 (22%) | **0.028** |
| Sulfalazine | 7 (9%) | 11 (3%) | **0.017** |
| Cyclosporin | 16 (21%) | 60 (15%) | 0.240 |
|  |  |  |  |

**Supplementary Table-8**: csDMARDs used ever in the difficult-to-treat (D2T) vs the non-D2T patients (main definition)

D2T (Difficult-to-treat), DMARDs (Disease modifying antirheumatic drugs), csDMARDs (Conventional synthetic DMARDs)

| **Features** | **D2T**  **(n=55)** | **Non-D2T**  **(n=673)** | **p-value** |
| --- | --- | --- | --- |
| Methotrexate | 45 (82%) | 586 (87%) | 0.300 |
| Leflunomide | 18 (33%) | 149 (22%) | 0.094 |
| Sulfalazine | 1 (2%) | 30 (4%) | 0.723 |
| Cyclosporin | 10 (18%) | 127 (19%) | 1.000 |
|  |  |  |  |

**Supplementary Table-9**: csDMARDs used ever in the difficult-to-treat (D2T) vs the non-D2T patients (MODA definition)

D2T (Difficult-to-treat), DMARDs (Disease modifying antirheumatic drugs), csDMARDs (Conventional synthetic DMARDs)

| **Features** | **D2T**  **(n=49)** | **Non-D2T**  **(n=344)** | **p-value** |
| --- | --- | --- | --- |
| Methotrexate | 38 (78%) | 295 (86%) | 0.140 |
| Leflunomide | 15 (31%) | 76 (22%) | 0.206 |
| Sulfalazine | 2 (4%) | 15 (4%) | 1.000 |
| Cyclosporin | 6 (12%) | 56 (16%) | 0.537 |
|  |  |  |  |

**Supplementary Table-10**: csDMARDs used ever in the difficult-to-treat (D2T) vs the non-D2T patients (MDA definition)

D2T (Difficult-to-treat), DMARDs (Disease modifying antirheumatic drugs), csDMARDs (Conventional synthetic DMARDs)
